# The Relationship between Adherence to the Mediterranean Diet, Intake of Specific Foods and Depression in an Adult Population (45–75 Years) in Primary Health Care. A Cross-Sectional Descriptive Study

**DOI:** 10.3390/nu13082724

**Published:** 2021-08-07

**Authors:** Bárbara Oliván-Blázquez, Alejandra Aguilar-Latorre, Emma Motrico, Irene Gómez-Gómez, Edurne Zabaleta-del-Olmo, Sabela Couso-Viana, Ana Clavería, José A. Maderuelo-Fernandez, José Ignacio Recio-Rodríguez, Patricia Moreno-Peral, Marc Casajuana-Closas, Tomàs López-Jiménez, Bonaventura Bolíbar, Joan Llobera, Concepción Sarasa-Bosque, Álvaro Sanchez-Perez, Juan Ángel Bellón, Rosa Magallón-Botaya

**Affiliations:** 1 Department of Psychology and Sociology, University of Zaragoza, 50009 Zaragoza, Spain; bolivan@unizar.es (B.O.-B.); rosamaga@unizar.es (R.M.-B.); 2 Institute for Health Research Aragón (IIS Aragón), 50009 Zaragoza, Spain; 3 Prevention and Health Promotion Research Network (redIAPP), ISCIII, 28220 Madrid, Spain; emotrico@uloyola.es (E.M.); ezabaleta@idiapjgol.org (E.Z.-d.-O.); sabela.couso@iisgaliciasur.es (S.C.-V.); anaclaveriaf@gmail.com (A.C.); jmaderuelo@saludcastillayleon.es (J.A.M.-F.); donrecio@gmail.com (J.I.R.-R.); patriciamorenoperal@gmail.com (P.M.-P.); mcasajuana@idiapjgol.info (M.C.-C.); tlopez@idiapjgol.org (T.L.-J.); bbolibar@idiapjgol.org (B.B.); jllobera@ibsalut.es (J.L.); Alvaro.Sanchez@osakidetza.net (Á.S.-P.); jabellon@uma.es (J.Á.B.); 4 Department of Psychology, Universidad Loyola Andalucía, 41704 Seville, Spain; igomezg@uloyola.es; 5 Fundació Institut Universitari per a la Recerca a L’Atenció Primària de Salut Jordi Gol i Gurina (IDIAPJGol), 08007 Barcelona, Spain; 6 Atenció Primària Barcelona Ciutat, Gerència Territorial de Barcelona, Institut Català de la Salut, 08007 Barcelona, Spain; 7 Nursing Department, Faculty of Nursing, Universitat de Girona, 17004 Girona, Spain; 8 Primary Care Research Unit, Área de Vigo, SERGAS, 36201 Vigo, Spain; 9 I-Saúde Group, Galicia Sur Health Research Institute (IIS Galicia Sur), SERGAS VIGO, 36213 Vigo, Spain; 10 Unidad de Investigación en Atención Primaria de Salamanca (APISAL), 37005 Salamanca, Spain; 11 Instituto de Investigación Biomédica de Salamanca (IBSAL), 37007 Salamanca, Spain; 12 Gerencia de Atención Primaria de Salamanca, 37007 Salamanca, Spain; 13 Gerencia Regional de Salud de Castilla y León (SACyL), 37005 Salamanca, Spain; 14 Faculty of Nursing and Physiotherapy, University of Salamanca, 37008 Salamanca, Spain; 15 Biomedical Research Institute of Málaga (IBIMA), 29010 Málaga, Spain; 16 Faculty of Medicine, Universitat Autònoma de Barcelona, 08193 Cerdanyola del Vallès, Spain; 17 Servei de Salut de les Illes Balears, 07003 Palma de Mallorca, Spain; 18 Aragonese Healthcare Service (SALUD), 50009 Zaragoza, Spain; ctbosque@hotmail.com; 19 Primary Care Research Unit of Bizkaia, Deputy Directorate of Healthcare Assistance BioCruces Bizkaia Health Research Institute, Basque Healthcare Service—Osakidetza, 48903 Barakaldo, Spain; 20 Department of Public Health and Psychiatry, University of Málaga, 29016 Málaga, Spain; 21 Department of Medicine, Psychiatry and Dermatology, University of Zaragoza, 50009 Zaragoza, Spain

**Keywords:** Mediterranean diet, depression, chronic diseases, cross-sectional study, primary healthcare

## Abstract

Background: The relationship between the quality of the diet and the adherence to the Mediterranean diet with the presence of persistent or recurrent depressive symptoms have been described. The objective of this study is to analyze the relationship between adherence to the Mediterranean diet and the intake of specific foods in primary care patients aged 45 to 75, having subclinical or major depression. The study also specifically analyzes this relationship in individuals suffering from chronic diseases. Methods: A cross-sectional descriptive study was conducted. 3062 subjects met the inclusion criteria from the EIRA study. Sociodemographic variables, clinical morbidity, depression symptomatology (PHQ-9) and adherence to Mediterranean diet (MEDAS) were collected. Results: Being female, younger, with a higher BMI, consuming more than 1 serving of red meat a day and drinking more than one carbonated or sugary drink daily, not consuming 3 servings of nuts a week and not eating 2 vegetables cooked in olive oil a week are predictors of having higher depressive symptomatology. Conclusions: Assessing the type of diet of patients presenting depressive symptoms and promoting adherence to a healthy diet is important, especially in patients with chronic diseases. However, depression is a very complex issue and the relationship between nutrition and depression must be further examined.

## 1. Introduction

Depression is considered to be the leading cause of disability worldwide, contributing to the overall global burden of morbidity and mortality. By 2030, it will most likely be the main contributor to morbidity [1]. According to the Global Burden of Disease Study (GBD), depression is the third cause in women and the fifth in men of years lived with disability [2]. It is also associated with the presence of chronic diseases [3,4,5,6,7], that are very prevalent in Western societies [8]. The onset and maintenance of depression have been related to a wide variety of biological and psychosocial factors, many of which relate to different aspects of lifestyles, particularly, nutrition [9,10,11,12,13,14,15,16]. This association between nutrition and depression appears to be consistent across countries, cultures and populations [17].

Numerous studies have described the relationship between obesity and depression [18], and between the quality of the diet and the presence of persistent or recurrent depressive symptoms [17,19]. A poor diet (defined in different studies by factors such as an abundance of saturated fats, “trans” fats, sweets, fast food, high sodium/potassium ratio, few vegetables and fish) is associated with recurrent depressive symptoms [20,21,22]. This association has been reliably confirmed, from basic research with animal models [23,24,25], to population studies providing the highest levels of evidence according to the scientific method [26,27]. An inadequate diet has been associated with the onset of depression and the higher severity of its symptoms. Therefore, more severe cases of depressions have been associated with more deficient diets [28,29]. On the other hand, various studies have suggested that a healthy diet is inversely related to depressive symptoms, according to a dose-response effect [30,31]. Therefore, it may be concluded that a low-quality diet is an avoidable risk factor for depression, supporting the possibility of using diet as an adjuvant treatment [20].

One of the diet patterns that has been studied with regard to the prevention of depression is the Mediterranean diet [17,19,32]. It consists of a predominant consumption of fruits, nuts, vegetables, cereals (especially in the form of bread), legumes, fish (more than meats), limited portions of meat, primarily white meat (such as chicken, turkey or rabbit); and a moderate quantity of red wine. The primary source of fat is olive oil while cheese is the primary source of dairy. Adherence to this dietary pattern ensures adequate nutrients such as antioxidants (especially olive polyphenols and wine resveratrol), selenium, B vitamins and omega-3 fatty acids [33]. This dietary pattern contains a balanced amount of fruit, vegetables, cereals, nuts, legumes and fish [19].

Basic research, for example, in experimental studies with rats, has revealed that the ongoing consumption of olive oil has neuroprotective effects, reducing behavioral alterations by acting on the metabolism of the serotonin and dopamine neurotransmitters. This supports its potential use as a therapeutic substance to treat depression and anxiety [24,25]. In elderly patients, it has also been found that the consumption of abundant vegetables, and more specifically, the Mediterranean-type diet [34] protects against depression. The consumption of olive oil is a predictor of fewer cases of diagnosed depression in the elderly [35]. This protective effect of the Mediterranean diet against the onset of depressive symptoms has also been demonstrated in middle-aged women, with a dose-response effect [36].

The PREDIMED study, using a Spanish population sample, revealed that adherence to the Mediterranean diet was associated with an improved concentration of brain-derived neurotrophic factor (BDNF) in depressed patients [37,38]. Some studies, however, have confirmed that there is currently a decline in the habit of following the Mediterranean diet in Spain and other Mediterranean countries, in favor of a more “Westernized” diet, rich in meat products, fried food, refined cereals, sugary drinks and processed foods, and lacking fresh fruits [39]. Multiple factors have been identified as predictors of poor adherence to the Mediterranean diet, including female sex, obesity and diabetes [40,41]. Studies should be carried out to determine the relationship between knowledge and actual practice, for example, at a primary healthcare level.

The objective of this study is to analyze the relationship between adherence to the Mediterranean diet and the intake of specific foods (fruits, nuts, vegetables, cereals, legumes, fish, meat and wine) in primary care patients aged 45 to 75, having subclinical or major depression. The study also specifically analyzes this relationship in individuals suffering from chronic diseases.

## 2. Materials and Methods

### 2.1. Design

A cross-sectional descriptive study was conducted, as part of the EIRA study [42]. This study had the main objective of evaluating the effectiveness, cost-effectiveness and implementation strategy of a complex multiple risk intervention to promote healthy behaviors in individuals aged 45 to 75 who were patients at Primary Healthcare Centers (PHCs). The EIRA methodology consisted of a randomized, controlled, hybrid type-2 trial using two parallel groups.

### 2.2. Study Population

The participating population consisted of 3062 subjects meeting the following inclusion/exclusion criteria from the EIRA study:

The following inclusion criteria were used: Individuals aged 45 to 75 years old, with two or more unhealthy behaviors (tobacco use, poor adherence to the Mediterranean diet, insufficient physical activity, cardiovascular risk and/or risk of depression) seen at the participating PHCs. In addition, they had to have an assigned doctor and nurse from the health center and their participation was voluntary. The exclusion criteria were: (1) individuals with serious, advanced illnesses, cognitive impairment, dependence, severe mental illness, participating in a home care program and with active or terminal cancer treatment, (2) individuals who would not be in Spain during the study period and (3) individuals who did not understand written and/or spoken Spanish.

Study participants were recruited from 26 PHCs in seven of the 17 Spanish autonomous communities: Andalusia, Aragon, the Balearic Islands, the Basque Country, Castile and León, Catalonia and Galicia. Offering universal coverage with free access to all citizens, the Spanish health system is funded by public sources and depends predominantly on the public sector.

### 2.3. Sample Size

This study’s sample size corresponds to that calculated for the EIRA study, which anticipated a difference in the percentage of individuals revealing a positive change of at least 8% between the two groups in one or more of the three behaviors. Assuming a 30% patient loss to follow-up, an alpha risk of 5%, a beta risk of 20% and an intra-cluster correlation of 0.01, it was considered necessary to study a minimum of 140 participants for each PHC, a total of 3640 individuals (1820 for each of the two groups, 13 PHCs per group) [42]. A total of 4387 participants were evaluated for eligibility, of which 532 did not provide consent, 333 engaged in only one unhealthy behavior and 460 did not attend the baseline assessment visit. Finally, 3062 participants were included in the study.

Participant selection was made amongst the subjects who attended the consultation of a family doctor or nurse at the participating PHCs for any reason and who met the inclusion criteria, considering age and gender quotas. The recruitment period was 12 months, beginning in January of 2017.

### 2.4. Study Variables

The following variables were collected for this study:

Sociodemographic variables: The variables of sex, age, place of birth, marital status (married or living with a partner, not married nor living with a partner), work activity (student, active worker, unemployed, housewife/househusband, retiree, others as temporary or permanent work disability) and educational level (primary or lower education, secondary or higher education) were considered.

The main variable is the intensity of the depressive symptomatology measured with the PHQ-9 questionnaire [43], using the validated Spanish version of this instrument [44]. It is one of the most widely used questionnaires for assessing depression in pharmacological and psychological studies. It is a short and self-applied scale intended to assist in diagnosing depression (DSM-IV criteria) and determining its severity. It is also useful for monitoring changes experienced by the patient over time. It has shown to have high reliability for screening depressive episodes, and the proposed cut-off point is 10 points [45].

Clinical morbidity variables: data regarding if the subjects had another chronic disease were collected (yes/no). The following were considered: cardiovascular diseases (myocardial infarction, heart failure, cardiovascular disease, hypertension and cerebrovascular disease), dementia, COPD, connective tissue disease, mild liver disease, diabetes, chronic kidney disease, cancer (in treatment and non-treatment), AIDS and osteoporosis. Weight, size, abdominal perimeter and BMI were also collected and established with the Charlson Comorbidity Index (CCI) [46] of the participating subjects.

Adherence to the Mediterranean diet: This variable was evaluated through the Mediterranean Diet Adherence Screener (MEDAS) test, which is a validated instrument for the rapid estimation of the patient’s adherence to the Mediterranean diet. Its usefulness in clinical practice has been recognized [47]. The MEDAS test was developed to quantitatively estimate the patient’s level of adherence to the cardio-protective characteristics of the traditional Mediterranean diet, quickly and effectively. It consists of 14 items, including food consumption and intake habits: the use of olive oil as the primary source of cooking fat, a preference for white meat over red meat, vegetable servings, portions of fruit, red meat or sausages, servings of animal fat, sugar-sweetened beverages, red wine, legumes, fish, commercial pastries and dressing food with a traditional sauce made of tomatoes, garlic, onion or leeks cooked in olive oil. The total score ranges from 0 to 14, with a higher score indicating better accordance with the Mediterranean diet. Participants with an inadequate or adequate diet were classified, depending on whether their score was less or greater than 9 [48].

Evaluators received specific face-to-face training to ensure the standardization of data collection.

### 2.5. Statistical Analysis

Given the large sample size, parametric tests were deemed appropriate, since in large samples, even when data distribution is not normal, the statistics tend to be normal [49]. First, a descriptive analysis of the sample was performed to obtain the mean and standard deviation for the quantitative variables and the frequency and percentages for the qualitative ones. This descriptive analysis was also performed for the chronic disease variable (yes/no), comparing these groups using the Student’s *t*-test or Chi-square test. To analyze the relationship between the PHQ-9 score and the sociodemographic variables and adherence to the Mediterranean diet (both in general and item by item), a correlation between the quantitative variables was performed using the Pearson Correlation Coefficient, and a comparison group analysis was performed for the qualitative variables using the Student’s *t*-test. Finally, a multiple linear regression was performed, controlling for the influence of the various independent variables [50]. The educational level variable was categorized into two categories to be included in the multiple regression. All of the covariates were introduced in the regression models using a stepwise method [51] to obtain a better fitting result upon statistical analysis. These analyses were performed considering the entire sample and were compared with those of individuals with any comorbidity (yes/no).

Data from the questionnaire were statistically analyzed using the SPSS v.25 [52] and AMOS v.20 statistical packages. All significance levels were established at 0.05. Observations having any missing data were eliminated.

### 2.6. Ethics

The phase III protocol of the EIRA study was approved by the Clinical Research Ethics Committee (CREC) of the Primary Care Research Institute (IDIAP) Jordi Gol of Barcelona and the CREC of each of the seven participating autonomous communities. The study was performed in accordance with the national and international standards of the Helsinki and Tokyo Declarations. To be included in the study, patients had to provide their signed informed consent.

## 3. Results

Of the 3062 subjects participating in the study, 45.1% were men and 54.9% were women, with an average age of 58.03 (SD: 8.10). As seen in Table 1, the participant’s profile is that of a married individual who works (either as an employee or self-employed), suffering from one or more chronic diseases, having a low adherence to the Mediterranean diet and with an average score on the PHQ-9 questionnaire of 4.56 (SD: 5.007) out of 27 points. They did not tend to present depressive symptomatology. The comparison of sex emphasizes a significantly higher percentage of men having one or more chronic diseases and with a higher score on the CCI. A higher percentage of women, as well as individuals suffering from chronic diseases, were found to have subclinical depression or major depression, and also a higher score on the PHQ-9 questionnaire (men: 3.62 (SD: 4.42) vs. women: 5.33 (SD: 5.32), *p*-value < 0.001). It is also worth noting that participants suffering from chronic diseases were older (59.79 (SD: 7.98) vs. 55.39 (SD: 7.52), *p*-value < 0.001) and had a lower educational level. A higher percentage of these individuals were retired or had a permanent disability and had a higher BMI. As for adherence to the Mediterranean diet, a higher percentage of women displayed a good adherence (men: 16.5% vs. women: 19.5%; *p*-value = 0.034), with a higher MEDAS questionnaire score (men: 6.57 (SD: 2.01) vs. women: 6.92 (SD: 1.93), *p*-value < 0.001).

Table 2 shows the results of the bivariate analysis of depressive symptomatology, sociodemographic variables (sex, age, educational level), BMI, adherence to the Mediterranean diet and the intake of specific foods. It is revealed that women had a significantly higher score on the PHQ-9 (men: 3.62 (SD: 4.42) vs. women 5.33 (SD: 5.32), *p*-value < 0.001), as well as younger people in the sample (−0.084, *p*-value < 0.001). The overall sample revealed a significant relationship between a higher BMI and a higher score on the PHQ-9 (0.042, *p*-value = 0.023), but this relationship does not appear when independently analyzing individuals either with or without chronic diseases. Those following a proper Mediterranean diet had significantly less depressive symptomatology (i.e., a lower score on the PHQ-9 questionnaire) (4.01 (SD 4.56) vs. 4.68 (SD 5.09), *p*-value = 0.003) and this result was also confirmed for the female sex and individuals without any chronic disease. A higher score obtained in the MEDAS questionnaire has been significantly related to a lower presence of depressive symptomatology (−0.073, *p*-value < 0.001). On the other hand, regarding the depressive symptomatology, it was found that individuals who usually cook with olive oil (4.49 (SD: 4.97) vs. 5.23 (SD: 5.29), *p*-value = 0.036), consume 3 or more servings of nuts a week (4.19 (SD: 4.68) vs. 4.68 (SD: 5.11), *p*-value = 0.014), eat less than one serving of red meat a day (4.30 (SD: 4.98) vs. 5.01 (SD: 5.01), *p*-value < 0.001), drink 7 glasses of wine a week (3.96 (SD: 4.56) vs. 4.71 (SD: 5.10), *p*-value < 0.001), or less than one carbonated or sugary drink a day (4.35 (SD: 4.78) vs. 5.09 (SD: 5.51), *p*-value < 0.001) have a less depressive symptomatology. This association has also been found for the female sex, except for wine consumption. Unexpectedly, it was found that consumption of two or more servings of vegetables a day is significantly related to increased depressive symptomatology (4.90 (SD: 5.02) vs. 4.44 (SD: 4.99), *p*-value = 0.029). This relationship has also been found for individuals with chronic diseases and those consuming 3 or more pieces of fruit a day.

As for the multivariate analysis, Table 3 shows that being female, younger, with a higher BMI, consuming more than 1 serving of red meat a day and drinking more than one carbonated or sugary drink daily, not consuming 3 servings of nuts a week and not eating 2 vegetables cooked in olive oil a week are predictors of having higher depressive symptomatology. This model explains 5% of the overall variance. As for the model obtained for individuals having a chronic disease, predictive factors are being female, younger, consuming more than one soft drink a day, the CCI, eating more than 3 pieces of fruit a day and not eating 3 servings of nuts a week. This model explains 6% of the overall variance. Furthermore, regarding the model obtained for individuals without a chronic disease, the predictive factors are being female, younger and consuming more than 1 serving of red meat a day. This model explains the best percentage of variance at 3.9%. 

## 4. Discussion

This study aims to examine the relationship between adherence to the Mediterranean diet and the intake of specific foods (fruits, nuts, vegetables, cereals, legumes, fish, meat and wine) in patients with subclinical or major depression in primary care. It also specifically analyzes this relationship in individuals with chronic diseases. The bivariate analysis reveals that individuals with a higher PHQ-9 score had an inadequate diet and a lower adherence to the Mediterranean diet. This significant relationship has also been found for individuals without chronic diseases, although, for those with a chronic disease, the relationship was almost significant (*p*-value = 0.051). Our results confirm past evidence that suggests that the severity of depression and current depression diagnosis are associated with an unhealthy dietary intake and poorer dietary quality, a higher intake of sweets and fast-food/savory snacks and a lower Mediterranean diet score [53].

The mechanisms involved may be linked to an enhanced production of BDNF, and therefore essential functions such as neuroplasticity, neuron survival and the growth and differentiation of new neurons and synapses [54,55]. A link has also been suggested between specific foods and a higher depressive symptomatology. However, despite this significant relationship, the R^2^ resulting from the multivariate analysis is small, indicating that depression is a complex entity involving various factors (biological, psychological, social, etc.) in its onset and maintenance [11,12,14,56,57]. This suggests the need for a comprehensive approach based on different considerations. Scientific evidence on the relationship between specific nutrients and depression is not yet conclusive [58,59,60,61,62].

As for sex, a significant relationship has been found in both the bivariate and multivariate analyses, since being female is associated with having a more significant depressive symptomatology. This result is coincidental and has been confirmed in numerous studies [63,64,65]. Regarding age, this study found that younger adults (under the age of 45) have higher depressive symptomatology. This can be explained since the risk of depressive disorder may decrease with age, as complex diagnostic screening questions may exaggerate lower rates of depression among older people [66]. It was also found that when controlling for variables such as sex, marital status, educational level, financial stress, chronic medical diseases, functional disability, cognitive problems, stressful life events, loneliness and social support, the age variable is not significant in depression [67]. If we consider that this study has been carried out in a sample of Spanish population, it has been found that in Spain, the mean age at first diagnosis for men is 49.89 (SD: 17.62) and 52.95 in women (SD: 17.10) [68]. Spain has one of the latest median ages of onset [69].

It has also been confirmed that individuals with chronic diseases present more significant depressive symptomatology, coinciding with abundant literature [3,4,5,6]. However, it is highlighted that adherence to the Mediterranean diet, as well as the intake of specific foods by individuals with and without diseases, has not been found to differentiate this symptomatology, with the exception of the consumption of fruit, wine and nuts, which is higher in those with chronic diseases. It was expected that individuals with chronic diseases would display a greater adherence to the Mediterranean diet to control their illness. A direct relationship has also been found in both the bivariate and multivariate analysis between BMI and depression, coinciding with various past studies [18,70].

Regarding adherence to the Mediterranean diet worldwide, a recent study shows that Southern Mediterranean countries (in which Spain is included), have a higher adherence comparing with the worldwide mean. However, Mediterranean countries altogether scored a lower adherence to the Mediterranean diet comparing with the worldwide mean [71].

When considering the intake of specific foods and their relationship with a lower presence of depressive symptomatology, the consumption of nuts, vegetables (cooked in sauce) and the use of olive oil are of special relevance in the bivariate and multivariate analyses. In addition, a higher consumption of sugary drinks and red meats has been found to be related to a higher presence of depressive symptomatology.

Nuts and olive oil (used for cooking and sautéing vegetables, as dressing) are rich in “polyunsaturated fatty acids” (PUFA), which are essential fatty acids. These fatty acids have a proven relevance in terms of depression disorder. The main brain PUFAs formed from these essential fatty acids are docosahexaenoic acid (DHA) and eicosapentaenoic acid (EPA), derived from omega-3 alpha-linolenic fatty acid, and docosatetraenoic acid (DTA), derived from omega-6 linoleic acid. Studies in depressed patients have shown that their DHA levels in adipose tissue were significantly lower than those of non-depressed patients, up to −34.6%. Moreover, maintaining adequate levels of DHA has been found to be inversely related to depression [72,73,74]. DHA and EPA deficiency could be associated with mood disturbance, somatic symptoms, cognitive dysfunction and other concurrent diseases in depressed individuals [75]. In elderly patients, interventions intended to enrich their diets through an increased intake of DHA and EPA correlate with an improvement in depressive symptoms and a better self-perception of physical health [76]. Regarding the essential fatty acid food source, bluefish, especially wild ones, are the highest in omega-3. Nuts (especially nuts and almonds) and other vegetable oils are also rich in omega-3 fatty acid, with the highest quality being found in extra-virgin olive oil [19]. In our study, fish consumption has not been found to have a significant relationship with depressive symptomatology, possibly due to the sample’s low adherence to fish consumption (approximately 35% of the sample consumes 3 servings of fish or seafood a week) and no specification of bluefish is established; therefore, the consumption of PUFAs is not explicitly valued through this type of fish.

Similar to vegetables and fruits, nuts also provide vitamins and minerals, mainly B vitamins, zinc and selenium. Mental health and well-being have been improved by increasing the consumption of fresh fruits and vegetables, which provide flavonols (kaempferol, isorhamnetin and myricetin) [77,78,79].

Regarding B vitamins, vitamin B12 deficiency has been linked to depression [19]. Vitamin B6, although possibly involved in the same sense, does not have a clearly established relationship with depression [19,80]. Some studies have described an association between increased vitamin B1 levels and improved mood [81]. As for vitamin B9, also called folate or folic acid, its deficit has also been shown to increase the risk of depression. Thus, in an 11-year follow-up study on middle-aged Finnish men, below-average folic acid intake led to a risk of developing depression that was more than three times higher [82]. This relationship has been confirmed in a study by Sánchez-Villegas et al. [19]. On the other hand, folic acid deficiency is more common in individuals with depression [83]. It has also been found to reduce the patient’s response to antidepressants [26].

Other nutrients, such as zinc, are associated with lower levels of this disorder [83]. Zinc supplements added to antidepressant treatment have been found to further improve symptoms, with this phenomenon being shown in females [84].

Selenium is another essential micronutrient whose deficit has been linked to the development of major depression in women; the prevalence of which has been found to be over two times that of women with an adequate intake [85]. The recommended daily intake to optimize plasma selenoprotein P concentration is approximately 100 micrograms a day, with the current average intake in the West being about half of this [86]. Excessive supplementation could be detrimental as it may increase the risk of type-2 diabetes [87]. It is present in the Mediterranean diet in adequate amounts [33,88].

Even though fruits also provide vitamins and minerals, our study has found that individuals with chronic diseases who consume 3 or more pieces of fruit a day have a higher PHQ-9 score. This may be explained by the fact that the MEDAS questionnaire does not discriminate between types of fruits consumed. Studies have confirmed that eating an apple a day has positive effects on mood [89], but other fruits having high levels of fructose have depressive effects on both animal models [90] and human models [91].

Even though the alcohol-depression relationship is well established in the literature and recent studies have associated multiple alcohol-related dietary habits (among them, the consumption of red wine) with depression [92], in this study, the consumption of red wine has been associated with lower levels of depression. This could be due to the high level of resveratrol found in red wine, which may be deemed an effective treatment for depression in animals [93]. Given that recent studies in humans are limited, future research should focus on resveratrol’s effects on depression in individuals to determine whether or not it may be used as a natural antidepressant with fewer adverse effects.

On the other hand, analyses carried out in all population groups show that the consumption of red meat is related to a higher presence of depressive symptomatology. Even though red meat also contains vitamins and minerals with a positive effect on mood, an excessive consumption has been found to be a risk factor for depression [94]. Meat rich in saturated fatty acids and red and/or processed meat are associated with an altered hypothalamic-pituitary-adrenal axis. Moreover, it should be considered that a high intake of fatty and processed foods is correlated with pro-inflammatory activity, having a detrimental effect on the cardiovascular system. This increases the risk of depression if the microvascular dysfunction is located in the brain [94].

An excessive consumption of sugary drinks has also been shown to be related with more significant depressive symptomatology. Sugars have been shown to impact very negatively on brain proteins, mainly on neutrophins, which are known to play a relevant role in depression since they protect the brain against oxidative stress and promote the growth of new brain cells [95].

This study has strengths as well as limitations. Among its strengths, the sample stands out, for both its size and its origin. Furthermore, this study analyses the depressive symptomatology with regard to adherence to the Mediterranean diet and the intake of specific foods, providing evidence for both nutrients from food and the Mediterranean diet’s food synergy. 

The study’s design may be considered a limitation since cross-sectional methodology does not establish causality relationships. Furthermore, having such a large sample implies that significant differences can be obtained without translating into large differences at the clinical level. However, these results may clarify aspects of depression, since it is a complex pathology both in its ethology and in treatment. Another study limitation is the instrument used, which, although quick and easy-to-administer and very useful in measuring adherence to the Mediterranean diet, does not permit discrimination between types of fish, fruit, etc. Therefore, it is impossible to differentiate the type of food consumed and the nutrients found. Moreover, the MEDAS questionnaire does not consider eating behavior but rather, it only measures the quantity and frequency of food consumption. In addition, we measure depression with the PHQ-9, and we base the differences in the severity of depression on its numerical result

## 5. Conclusions

Adherence to the Mediterranean diet and the resulting consumption of nuts, vegetables and olive oil has been found to relate to a lower presence of depressive symptomatology. On the other hand, a poorer adherence to the Mediterranean diet and an excessive consumption of sugary drinks and red meats has been related to higher depressive symptomatology. Assessing the dietary pattern of patients presenting depressive symptoms and chronic diseases and promoting adherence to a healthy diet could be important, as well as assessing the depressive symptoms or chronic conditions of people with unhealthy dietary patterns. However, depression is a very complex issue and the relationship between nutrition and depression must be further examined to obtain additional scientific evidence.

## Figures and Tables

**Table 1:** Description of the sample in terms of sociodemographic variables, presence of chronic diseases, depression and adherence to the Mediterranean diet. Comparison according to the presence of chronic diseases.

Variables		N (%) Mean (SD)	Presence of Chronic Diseases		
			Yes N = 1795	No N = 1043	*p*-Value
SEX	Males	1381 (45.1%)	887 (49.4%)	400 (38.4%)	<0.001
	Females	1681 (54.9%)	908 (50.6%)	643 (61.6%)	
	AGE	58.03 (8.10)	59.79 (7.98)	55.39 (7.52)	<0.001 *
	45–54 range	1186 (38.7%)	528 (29.4%)	546 (52.3%)	
	55–64 range	1077 (35.2%)	673 (37.5%)	331 (31.7%)	<0.001
	65–75 range	799 (26.1%)	594 (33.1%)	166 (15.9%)	
MARITAL STATUS	Single	341 (11.2%)	187 (10.4%)	130 (12.5%)	
	Married or couple	2079 (68.5%)	1232 (68.8%)	705 (68%)	0.057
	Separated or divorced	413 (13.6%)	236 (13.2%)	148 (14.3%)	
	Widower	201 (6.6%)	135 (7.5%)	53 (5.1%)	
	Other	2 (0.1%)	1 (0.1%)	1 (0.1%)	
EDUCATIONAL LEVEL	Higher education	514 (16.9%)	(14.1%)	226 (21.8%)	
	Secondary studies	1194 (39.4%)	665 (37.2%)	446 (43%)	<0.001
	Primary studies	1146 (37.8%)	743 (41.5%)	326 (31.4%)	
	No studies	179 (5.9%)	129 (7.2%)	39 (3.8%)	
WORK ACTIVITY	Student	8 (0.3%)	3 (0.2%)	4 (0.4%)	
	Actively employed	1374 (45.3%)	663 (37.1%)	605 (58.4%)	
	Temporary work disability	82 (2.7%)	55 (3.1%)	25 (2.4%)	<0.001
	Unemployed	286 (9.4%)	164 (9.2%)	95 (9.2%)	
	Housewife/househusband	368 (12.1%)	234 (13.1%)	109 (10.5%)	
	Permanent disability	111 (3.7%)	86 (4.8%)	19 (1.8%)	
	Retired	802 (26.5%)	583 (32.6%)	179 (17.3%)	
COUNTRY OF BIRTH	Spain	2848 (93.9%)	1689 (94.1%)	963 (93%)	
	Rest of Europe	49 (1.6%)	31 (1.7%)	16 (1.5%)	0.303
	America	116 (3.8%)	58 (3.2%)	48 (4.6%)	
	Asia	1 (0.0%)	1 (0.1%)	0 (0%)	
	Africa	20 (0.7%)	11(0.6%)	9 (0.9%)	
	CHRONIC DISEASE (Yes %)	1795 (63.2%)			
	Cardiopathy	125 (4.1%)			
	Vascular disease	94 (3.1%)			
	Cerebrovascular disease	45 (1.5%)			
	Hypertension	1197 (39.4%)			
	Dementia	2 (0.1%)			
	COPD	125 (4.1%)			
	Connective tissue disease	117 (3.9%)			
	Liver disease	78 (2.6%)			
	Diabetes	600 (19.7%)			
	Chronic kidney disease	32 (1.1%)			
	Cancer	130 (4.3%)			
	AIDS	5 (0.2)			
	Osteoporosis	94 (3.1%)			
	CCI *	2.88 (1.40)			
ANTHROPOMETRIC VARIABLES	Weight *	80.46 (17.69)	82.89 (18.31)	76.63 (16.34)	<0.001 *
	Abdominal perimeter *	100.87 (14.72)	103.42 (14.76)	96.72 (14.11)	<0.001 *
	BMI *	29.91 (5.78)	30.81 (5.83)	28.45 (5.42)	<0.001 *
	PHQ-9 score *	4.56 (5.007)	4.76 (5.21)	4.06 (4.58)	<0.001
	Subclinical and major depression (Yes %)	508 (16.6%)	308 (17.2%)	143 (13.7%)	0.015
	ADHERENCE TO DIET (Yes %)	555 (18.2%)	323 (18%)	207 (19.9%)	0.226
	Olive oil for cooking (Yes %)	2795 (91.5%)	1635 (91.2%)	966 (92.7%)	0.170
	+4 Tablespoons olive oil per day	1703 (55.7%)	995 (55.5%)	587 (56.3%)	0.676
	+2 Servings of vegetables per day	802 (26.2%)	462 (25.8%)	281 (27%)	0.489
	+3 Fruit per day	762 (24.9%)	478 (26.7%)	226 (21.7%)	0.003
	−1 Red meat per day	1961 (64.2%)	1194 (66.6%)	662 (63.5%)	0.094
	−1 Butter or cream per day	2509 (82.1%)	1460 (81.5%)	877 (84.2%)	0.069
	−1 Sugary drinks per day	2212 (72.4%)	1292 (72.1%)	766 (73.5%)	0.416
	+7 Wine per week	610 (20%)	387 (21.6%)	176 (16.9%)	0.002
	+3 Legumes per week	636 (20.8%)	369 (20.6%)	230 (22.1%)	0.352
	+3 Fish-seafood per week	1081 (35.4%)	618 (34.5%)	374 (35.9%)	0.449
	−2 Pastries per week	1471 (48.1%)	874 (48.8%)	519 (49.8%)	0.595
	+3 Nuts per week	792 (25.9%)	428 (23.9%)	309 (29.7%)	0.001
	Preferable white meat	1803 (59%)	1095 (61.1%)	632 (60.7%)	0.812
	+2 Vegetables cooked in olive oil per week	1539 (50.4%)	892 (49.8%)	553 (53.1%)	0.091
	Total MEDAS rating *	6.77 (1.98)	6.80 (1.95)	6.87 (2.0)	0.344 *

Note: CCI, Charlson Comorbidity Index. BMI, Body Mass Index. Statistical used: Chi-square test and Student *t*-test *.

**Table 2:** Bivariate analysis between sociodemographic variables, MEDAS questionnaire items and depressive symptomatology (PHQ-9).

	Items MEDAS	PHQ-9	PHQ-9 Score by Presence of Chronic Diseases	
			Yes	No
SEX	Man	3.62 (4.42)	3.78 (4.69)	3.31 (3.92)
	Woman	5.33 (5.32)	5.72 (5.53)	4.54 (4.89)
	*p*-value	<0.001	<0.001	<0.001
AGE	Pearson	−0.084	−0.116	−0.091
	*p*-value	<0.001	<0.001	0.003
EDUCATIONAL LEVEL	Secondary and higher education	4.53 (5.04)	4.79 (5.27)	4.13 (4.71)
	Primary studies/non-studies	4.59 (4.97)	4.72 (5.16)	3.94 (4.34)
	*p*-value	0.741	0.777	0.535
BMI	Pearson	0.042	0.026	0.039
	*p*-value	0.023	0.269	0.209
Olive oil for cooking	Yes	4.49 (4.97)	4.69 (5.18)	4.04 (4.57)
	No	5.23 (5.29)	5.41 (5.55)	4.38 (4.64)
	*p*-value	0.036	0.128	0.547
+4 Tablespoons olive oil per day	Yes	4.58 (5.13)	4.78 (5.33)	4.04 (4.63)
	No	4.52 (4.84)	4.72 (5.07)	4.09 (4.52)
	*p*-value	0.734	0.824	0.854
+2 Servings of vegetables per day	Yes	4.90 (5.02)	5.27 (5.23)	4.15 (4.36)
	No	4.44 (4.99)	4.58 (5.20)	4.03 (4.66)
	*p*-value	0.029	0.018	0.697
+3 Fruit per day	Yes	4.79 (5.13)	5.22 (5.49)	3.86 (4.36)
	No	4.48 (4.96)	4.58 (5.11)	4.12 (4.64)
	*p*-value	0.145	0.029	0.439
−1 Red meat per day	Yes	4.30 (4.98)	4.61 (5.29)	3.69 (4.36)
	No	5.01 (5.01)	5.05 (5.05)	4.72 (4.86)
	*p*-value	<0.001	0.092	0.001
−1 Butter or cream day	Yes	4.48 (5.02)	4.68 (5.24)	3.96 (4.56)
	No	4.91 (4.93)	5.08 (5.12)	4.63 (4.65)
	*p*-value	0.074	0.212	0.09
−1 Sugary drinks per day	Yes	4.35 (4.78)	4.53 (4.97)	3.90 (4.39)
	No	5.09 (5.51)	5.35 (5.77)	4.51 (5.03)
	*p*-value	0.001	0.006	0.067
+7 Wine per week	Yes	3.96 (4.56)	3.94 (4.65)	3.83 (4.62)
	No	4.71 (5.10)	4.98 (5.34)	4.11 (4.57)
	*p*-value	<0.001	0.001	0.459
+3 Legumes per week	Yes	4.31 (5.05)	4.67 (5.33)	3.71 (4.76)
	No	4.62 (4.99)	4.78 (5.19)	4.16 (4.52)
	*p*-value	0.173	0.726	0.203
+3 Fish-seafood per week	Yes	4.49 (4.87)	4.86 (5.19)	3.74 (4.27)
	No	4.59 (5.08)	4.69 (5.23)	4.24 (4.73)
	*p*-value	0.583	0.522	0.083
−2 Commercial pastries per week	Yes	4.39 (5.09)	4.63 (5.28)	3.86 (4.73)
	No	4.71 (4.92)	4.87 (5.15)	4.27 (4.42)
	*p*-value	0.09	0.319	0.153
+3 Nuts per week	Yes	4.19 (4.68)	4.32 (4.85)	3.92 (4.46)
	No	4.68 (5.11)	4.89 (5.32)	4.12 (4.63)
	*p*-value	0.014	0.042	0.520
Preferably white meat	Yes	4.54 (5.02)	4.81 (5.27)	4.01 (4.50)
	No	4.57 (4.98)	4.66 (5.13)	4.14 (4.70)
	*p*-value	0.874	0.559	0.660
+2 Vegetables cooked in olive oil per week	Yes	4.41 (4.75)	4.60 (4.99)	4.03 (4.36)
	No	4.70 (5.24)	4.90 (5.43)	4.11 (4.81)
	*p*-value	0.114	0.232	0.785
Proper diet	Yes	4.01 (4.56)	4.51 (4.90)	3.11 (3.88)
	No	4.68 (5.09)	4.81 (5.28)	4.30 (4.70)
	*p*-value	0.003	0.343	0.001
Total MEDAS rating *	Pearson	−0.073	−0.047	−0.102
	*p*-value	<0.001	0.051	0.001

Note: BMI, Body Mass Index. Statistical used: Chi-Squared and Pearson *.

**Table 3:** Variant regression model of the PHQ-9 score and the variables of sex, age, educational level, BMI and MEDAS items. CCI in the individuals with chronic diseases.

PHQ-9 Score Global Sample	Coefficient	*p*-Value	95% Confidence Interval	
			Lower	Upper
Constant	4.521	<0.001	2.801	6.242
Sex (women vs. men)	1.859	<0.001	1.502	2.216
−1 Red meat per day (Yes vs. No)	−0.735	<0.001	−1.109	−0.361
Age (in years)	−0.045	<0.001	−0.067	−0.023
−1 Sugary drinks per day (Yes vs. No)	−0.710	0.001	−1.112	−0.308
BMI	0.036	0.023	0.005	0.067
+3 Nuts per week (Yes vs. No)	−0.423	0.040	−0.828	−0.018
+2 Vegetables cooked in olive oil per week	−0.360	0.047	−0.715	−0.005
R^2^	0.051			
R^2^ adjusted	0.049			
PHQ-9 score With chronic disease	Coefficient	*p*-value	95% Confidence interval	
			Lower	Upper
Constant	7.749	<0.001	5.683	9.815
Sex (women vs. men)	2.010	<0.001	1.527	2.494
Age (in years)	−0.108	<0.001	−0.147	−0.069
−1 Sugary drinks per day (Yes vs. No)	−0.829	0.002	−1.364	−0.293
CCI	0.291	0.008	0.078	0.505
+3 Fruit per day (Yes vs. No)	0.665	0.017	0.118	1.213
+3 Nuts per week (Yes vs. No)	−0.586	0.040	−1.147	−0.026
R^2^	0.062			
R^2^ adjusted	0.059			
PHQ-9 score Without chronic disease	Coefficient	*p*-value	95% Confidence interval	
			Lower	Upper
Constant	5.425	<0.001	3.188	7.662
Sex (women vs. men)	1.352	<0.001	0.779	1.925
−1 Red meat per day (Yes vs. No)	−1.073	<0.001	−1.656	−0.489
Age (in years)	−0.051	0.007	−0.089	−0.014
R^2^	0.039			
R^2^ adjusted	0.036			

Note: CCI, Charlson Comorbidity Index.

## Data Availability

The data presented in this study are available on request from the corresponding author. The data are not publicly available due to authors are still working with the data.
